# Association between Air Pollution and Emergency Room Visits for Atrial Fibrillation

**DOI:** 10.3390/ijerph14060661

**Published:** 2017-06-20

**Authors:** Angelo G. Solimini, Matteo Renzi

**Affiliations:** 1 Department of Public Health and Infectious Diseases, Sapienza University of Rome, Piazza A. Moro 5, 00185 Roma, Italy; 2 Department of Epidemiology, Lazio Regional Health Service, ASL Rome 1, 00147 Rome, Italy; m.renzi@deplazio.it

**Keywords:** air pollution, atrial fibrillation, time series, cardiac arrhythmias

## Abstract

Despite the large prevalence in the population, possible factors responsible for the induction of atrial fibrillation (AF) events in susceptible individuals remain incompletely understood. We investigated the association between air pollution levels and emergency department admissions for AF in Rome. We conducted a 14 years’ time-series study to evaluate the association between the daily levels of air pollution (particulate matter, PM_10_ and PM_2.5_, and nitrogen dioxide, NO_2_) and the daily count of emergency accesses for AF (ICD-9 code: 427.31). We applied an over-dispersed conditional Poisson model to analyze the associations at different lags after controlling for time, influenza epidemics, holiday periods, temperature, and relative humidity. Additionally, we evaluated bi-pollutant models by including the other pollutant and the influence of several effect modifiers such as personal characteristics and pre-existing medical conditions. In the period of study, 79,892 individuals were admitted to the emergency departments of Rome hospitals because of AF (on average, 15.6 patients per day: min = 1, max = 36). Air pollution levels were associated with increased AF emergency visits within 24 h of exposure. Effect estimates ranged between 1.4% (0.7–2.3) for a 10 µg/m^3^ increase of PM_10_ to 3% (1.4–4.7) for a 10 µg/m^3^ increase of PM_2.5_ at lag 0–1 day. Those effects were higher in patients ≥75 years for all pollutants, male patients for PM_10_, and female patients for NO_2_. The presence of previous cardiovascular conditions, but not other effect modifiers, increase the pollution effects by 5–8% depending on the lag. This study found evidence that air pollution is associated with AF emergency visits in the short term.

## 1. Introduction

Epidemiological evidences of the association between air pollution and cardiovascular diseases (CVD) have been growing in the last few decades [1,2,3]. Pooled estimates of the percent increase in CVD mortality, derived from meta-analyses and multiple city studies, range between 0.6 and 1.4 for any 10 µg/m^3^ increment of particulate matter (PM_10_ and PM_2.5_) [4]. Similarly, CVD hospital admissions are reported to increase with the level of pollution [5,6]. A recent meta-analysis concluded that the risk of myocardial infarction (MI) increases significantly with all air pollutants screened, except for ozone [7]. In a comparative risk assessment, given its nature of unavoidable exposure, it is not surprising that the fraction of MI cases attributable to traffic is the highest among the known triggers of MI [8].

One of the plausible mechanisms to explain the causal link between exposure to air pollution and increased CVD mortality and hospital admissions is through an adverse effect of inhaled pollutants on the cardiac autonomic control [9,10]. Previous studies showed that PM_2.5_ exposure was associated with increased heart rate and decreased heart rate variability [11], both markers of autonomic dysfunction [12]. Impaired autonomic modulation may cause the onset of premature ventricular or atrial contractions [11], two common forms of arrhythmia. Additionally, early studies on patients with implantable cardioverter-defibrillators (ICDs) suggested that ventricular arrhythmias were the main causes of severe cardiovascular complications of exposure to air pollution [9].

Recent studies also suggested the possible association of air pollution exposure with atrial fibrillation (AF), the most frequent cardiac arrhythmia diagnosed in clinical practice [13]. The prevalence of AF in the general population increases with age, being around 1% or less in adults younger than 60 years and reaching >9% in patients aged 80 or older [14,15]. Projected estimates point to a >2 fold increase of the number of AF patients by the year 2050 in both the US [15] and Europe [14]. In addition to the altered cardiac autonomic control [16,17,18], AF onset may be triggered by inflammation and oxidative stress [19,20] and atrial pressure changes [21] which can all be linked to air pollution exposure [10].

In this paper, we hypothesized that the increase of air pollution levels is associated with increased AF emergency hospital visits. This hypothesis is assessed through the study of 14 years’ time series of daily AF hospital emergency visits and pollution levels registered in the city of Rome. Because the AF may result from the cumulative effect of exposure events of different intensities sustained in the past, depending on the specific pollutant and of the physiological mechanisms linking the exposure to the outcome, we explored immediate (within 1 day), delayed (2–5 days), or extended (cumulative effect of 0–5 days) response of AF emergency hospital visits to the increase of pollution levels after adjusting for several time-varying covariates. Additionally, we studied the influence of pre-existing medical conditions as effect modifiers.

## 2. Methods

### 2.1. Study Population

The Rome municipality extends for 1287.36 km^2^ and had a registered population of 2,612,068 inhabitants during the 2001 census. Anonymous records for patients ≥35 years, with a ICD-9 code 427.31 (atrial fibrillation) as the primary diagnosis, have been extracted from the administrative database recording all emergency visits in the period from 1 January 2001 to 31 December 2014, registered from 51 Emergency Departments of hospitals in Rome. Individuals residing outside the city boundaries were excluded from the population in order to increase the likelihood that exposure corresponded with the measured air pollution.

### 2.2. Environmental Data

Hourly nitrogen dioxide (NO_2_), and particulate matter with diameter of ≤10 µm (PM_10_) and of ≤2.5 µm (PM_2.5_) levels were obtained from official online databases (ARPA Lazio, Regional Agency of Environmental Protection of the Lazio region). All data referred to hourly measurements and passed the quality control of the agency. PM_2.5_ data were available only for the period from 2006 to 2014. Only monitors with 75% yearly data coverage were considered eligible [22] and suburban monitors were preferred to traffic-pollution monitors. Exposure was defined as the average of daily mean concentrations by three fixed eligible monitors. Missing values for each pollutant on a specific day and monitor were imputed with the average measurements of that pollutant for that day from the other monitors, weighted by the ratio of the yearly average at that monitor over the yearly average at the other monitoring stations for the same pollutant [23]. Average daily meteorological variables (temperature, barometric pressure, and dew-point temperature) at one station in central Rome were obtained from the Air Force monitoring network. We combined information from the air temperature and dew-point temperature to obtain an apparent temperature (AT) [24], an index of human discomfort, because it was demonstrated to have a stronger effect on health outcomes than the air temperature [25,26]. AT was calculated by the formula: AT = −2.653 + (0.994 × Ta) + (0.0153 × Td2), where Ta is the air temperature and Td is the dew point temperature [26].

### 2.3. Statistical Analysis

We performed a time-series analysis using conditional quasi-Poisson regression models, which is a flexible alternative to case-crossover design and provides comparable estimates to classic quasi-Poisson and case-crossover models [27], but allows for an important computational improvement of the time requested for statistical analysis. Time trend effects were controlled by inserting three-way interaction terms between the year, month, and day of the week. As we aimed at evaluating a possible association between the short-term concentration levels of pollutants and acute episodes of AF, we used moving averages of PM_10_, PM_2.5_, and NO_2_ at lags from 0 to 5 days. Following the current nomenclature, we defined immediate (from day 0 to day 1), delayed (from day 2 to day 5), and extended (from day 0 to day 5) effects to air pollution [28]. Besides the pollution and the time terms, predictor variables included in the model were apparent temperature (linear and quadratic terms), barometric pressure (linear and quadratic terms), and indicator variables for influenza epidemics, holiday periods, and a dummy for summer population decrease. In the exploratory analysis, models fitted with natural cubic splines with three degrees of freedom for apparent temperature did not have smaller values of the Akaike’s Information Criterion compared to models fitted with linear and quadratic terms. Following similar studies conducted in Rome [28,29], we adjusted the model for warm temperatures by calculating the apparent temperature above the median of the whole study period at lag 0–1 day and we adjusted for cold temperatures by calculating the apparent temperature below the median of the whole study period at lag 1–6 days. Two time-varying confounders were included in the analysis: influenza epidemics and holiday periods. Similar to other studies [30], we defined the population decrease during holidays as a 3 level variable assuming values “0” for working days, “1” for other non-working days like Labor Day, and “2” representing Christmas, Easter, and summer multiple days’ vacation periods. The influenza peak period was defined as a variable with two possible levels where “1” represents the 3 weeks when the influenza incidence is peaking, and “0” for all other weeks [30]. The main model was also stratified for sex and age (<75; ≥75 years).

We used an individual approach using a logistic regression model to assess a possible effect modification following the case-only approach [31,32] that is suitable for modifiers that do not vary (personal characteristics), or do vary slowly (pre-existing chronic medical conditions) with the exposure of interest (e.g., pollution levels). This approach has the advantage, over traditional analyses (e.g., including several interaction terms in the main Poisson model), of simplifying the model specification [33]. Those individual data were available only for a single hospital (Policlinico Umberto I) and for a restricted time period (2001–2010), which represented 16.8% of all AF emergency visits registered in the same period in hospitals with comparable levels of emergency service (level 2 surgery). We evaluated a possible effect modification for: sex, age-category (<75 and >75 years old), and pre-existing medical conditions defined as binary variables: diabetes (ICD-9: 250), cardiovascular diseases (previous heart failure ICD-9: 428; cardiac dysrhythmias ICD-9: 427; myocardial infarction ICD-9: 410; stroke ICD-9: 434), chronic respiratory disease (ICD-9: 490-492, 496), and other chronic diseases (including liver ICD-9: 571; kidneys ICD-9: 585; oncological ICD-9: 140-239 and neurodegenerative diseases ICD-9: 331). We fitted separate logistic regression models with PM_10_, PM_2.5_, and NO_2_ as predictors and the presence or absence of the hypothesized modifying condition as the dependent variable in the case data series. Moreover, we controlled for long-term and seasonal trends including a term for seasonal effect (sine and cosine terms with a 365.24-day period), as described in detail elsewhere [32]. The resulting coefficients provide an estimate of the change in the incremental effect of pollution in the group holding the modifying condition compared to others [33].

Finally, we produced a sensitivity analysis to assess the potential concomitant effects of multi-exposure through bi-pollutant models for PM_10_, PM_2.5_, and NO_2_. We included, in the models of the main analysis, the other pollutant in turn at the same cumulative lag. Additionally, the association between air pollution and AF emergency visits was also assessed by distributed lag non-linear models (DLNM) [34,35] using the DLNM R package [36]. DLNM describes simultaneous non-linear and delayed effects of air pollution on hospital emergency visits. We fitted a linear function for air pollution and a spline function with 2 knots for the lag.

The results are reported as percent variation in the occurrence of emergency visits per a 10 µg/m^3^ increase in PM_10_, PM_2.5_, and NO_2_. For the case-only analysis, we reported results as interaction rate ratios (IRRs) estimating the Risk Ratios (RRs) for AF occurrence per 10 µg/m^3^ increases of pollutant concentration in persons who held the medical condition compared to persons who did not have the medical condition.

All analyses were performed with R software [37].

## 3. Results

In total, during the period from 2001 to 2014, 79,892 individuals were admitted to emergency departments of Rome hospitals because of AF (on average, 15.6 patients per day: min = 1, max = 36; Table 1). Men represented 45.6% with a median age of 72 years (min 35, max = 100), while the median age of women was 74 years (min = 35, max = 104). Summary statistics for air pollution, temperature, and relative humidity are reported in Table 1. As expected, the mean daily levels of different pollutants were correlated (r Pearson PM_10_-NO_2_ = 0.60, PM_2.5_-PM_10_ = 0.88, PM_2.5_-NO_2_ = 0.68).

Table 2 presents the effect of air pollution on emergency visits for AF at different lags. Considering the whole sample, we found statistically significant associations between PM_10_, PM_2.5,_ NO_2,_ and AF visits at immediate lags, but not at delayed or extended lags. At immediate lags, the greatest effect estimate was observed for PM_2.5_ (2.95%; 95% CI: 1.35, 4.67%), followed by PM_10_ (1.44%; 95% CI: 0.65, 2.26%), and NO_2_ (1.19%; 95% CI: 0.27, 2.13%). Those associations were greater for 75 years old or older patients with an estimated effect of 5.01% (95% CI: 2.59, 7.74%) for PM_2.5_, 2.70% (95% CI: 1.42, 4.07%) for PM_10_, and 1.50 (95% CI: 0.03, 3.02%) for NO_2_. In this age group a significant effect of PM_2.5_ at extended lag was also present (3.43%; 95% CI: 0.42, 6.66%). Gender specific associations were generally greater for men, being significant for PM_10_ at immediate (1.51%; 95% CI: 0.15, 2.91%) and extended lags (1.87%; 95% CI: 0.15, 3.64%) and for NO_2_ at delayed (1.83%; 95% CI: 0.10, 3.62%) and extended lags (2.40%; 95% CI: 0.35, 4.55%). The only significant association between pollution levels and emergency visits for AF for women was with PM_2.5_ at immediate lag (3.51%; 95% CI: 1.29, 5.90%). In bi-pollutant models, the effects of PM_10_ and PM_2.5_ with NO_2_ in the model on AF visits were weaker but significant at immediate lag, while the effects of NO_2_ (with PM_10_ and PM_2.5_) were not significant at all lags investigated (Table S1). Coherently with the main analysis, the DLNM analysis resulted in effect coefficients of similar size at lag 0–1 day (Supplementary Figures S1–S3).

Table 3 shows the analysis of effect modification in the subset of patients with detailed medical records (see Supplementary Table S2). The effects of PM_10_ and NO_2_ (but not of PM_2.5_) on AF visits were 6–9% higher in individuals with previous CVD conditions (Table 3). All other pre-existing medical conditions were not significant modifiers of the pollution effects, although increasing patterns were apparent for chronic respiratory disease with NO_2_ (not significant effect increases of 3–6%, Table 3).

## 4. Discussion

Atrial fibrillation is the most common arrhythmia encountered in clinical practice and AF patients may present general symptomatology such as chest pain, palpitations, and dyspnea [38]. Although it has a high incidence in the population, the underlying mechanisms responsible for induction and perpetuation of AF remain incompletely understood [39]. Altered cardiac autonomic control, inflammation and oxidative stress, and atrial blood pressure changes are among the mechanisms suggested for AF onset [40]. We found that higher concentrations of air pollution may trigger AF emergency visits in the short term. The effect is particularly consistent with increasing PM_2.5_ concentration at 0–1 day lag, with effects ranging from nearly a 3% increase of hospital visits for AF for the whole sample to 5% for individuals ≥75 years. We also found significant effects at lag 0–1 day for PM_10_ and NO_2_. The results of the models stratified by age category confirmed the higher sensitivity of subjects ≥75 years of age as observed for hospitalizations for other cardiac causes, while the results stratified by sex were less consistent.

Epidemiological evidence of the air pollution effect on AF onset is contradictory. Studies on patients with implantable cardioverter-defibrillators reported increased AF risk with increasing ozone (O_3_) [41,42], NO_2_ [43], PM_2.5_ [44], and sulfate (SO_4_) [42]. However, no associations were also reported with PM_2.5_ [45], NO_2_, carbon monoxide (CO), sulphur dioxide (SO_2_) [45,46], black carbon, SO_4_, and O_3_ [46]. A recent study on individuals that referred to a 24 h period of ambulatory cardiac monitoring did not find a significant increase of AF events with NO_2_ and PM_2.5_, possibly because of the relative low levels of pollution in the study area [47]. Results of the association between AF and air pollution are also not consistent in population based studies. For example, Sade et al. examined 1458 hospital admissions for new-onset AF and found a significant association with CO, NO_2_, and SO_2_, but not with PM_10_ and O_3_ [48]. Similarly, a UK study examined >2 million of CVD emergency hospital admissions and found significant association of AF with NO_2_ but not CO, O_3_, PM_10_, PM_2.5_ and SO_2_ [49]. NO_2_ derives from direct emissions of diesel vehicles and from secondary reactions of ozone with nitric oxide and typically has a spatial correlation with other combustion products (such as exhaust particles, carbon monoxide, and sulphur dioxide) emitted at the same time [50]. Therefore, the mixture of pollutants derived from exposure to traffic (for which NO_2_ is a marker) or NO_2_ by itself, might lead to chronic autonomic dysfunction through multiple pathways [4], increasing the risk of AF episodes. Notably, in a large population time-series study in the US on more than 10,000 people, no association between cumulative exposure to PM_2.5_ and acute episodes of AF was found [51], although the omission in the statistical model of important covariates could partially explain the result [50]. The latter argument might also explain the absence of association between the acute onset of AF and air pollution observed in a short time series study conducted in Italy [52].

In the case-only analysis, we found an increase of risk of AF emergency admission for subjects that had a previous medical examination or treatment because of cardiovascular problems. We found statistically significant results for PM_10_ and NO_2_ and marginally not significant results for PM_2.5_, possibly because of the low power used to detect a significant effect for the latter pollutant for which the monitoring data availability started in 2006. It is well documented that patients with previous episodes of major CVD events or that are suffering from chronic CVD have a higher probability of being re-hospitalized for the same or another cardiac complication [53]. In our analysis, up to 75% of individuals had prior history of CVDs and thus these individuals are at particularly high risk for AF, which may predict more serious and potentially life-threatening events such as stroke [38]. For example, a time-series study in Montreal conducted on more than 150,000 deaths of >65 years people, found a positive association between NO_2_ and PM_2.5_ and mortality percent change in individuals with a previous diagnosis of AF [54]. In a similar study in UK, among all pollutants, only PM_2.5_ was associated to AF as the primary cause of death in a large event database [49]. Also, an analysis of long term exposure to high ambient NO_2_ pointed to a higher susceptibility of subjects with an underlying cardiovascular disease [55].

As in many other time-series studies, several study limitations need to be addressed. First, our study assessed the association of AF with air pollution considering the date of emergency visits that may come after the time of AF onset by hours or days, potentially causing exposure misclassification. Another possible limitation that we have to point out relates to the information provided by the health regional databases using ICD-9 codes, so we cannot use a more modern and accurate classification such as ICD-10. However, we considered that a possible information bias due to less accuracy in ICD-9 codes could be non-differential, and therefore was not able to alter the estimates of association.

Since studies with ICDs have pointed out a very rapid response of AF to increases of pollution levels [46], by using emergency care patients and examining different short-term time lags, we possibly reduced the misclassification due to the different timing between the pollutant and the case series. Second, temporal variation of exposure was estimated through the time-series of pollution levels derived from within-city monitor measures, excluding surrounding towns and villages. Like in other studies, we could not apply individual exposures for cases, so we used the daily average levels from all air quality monitors with sufficient temporal coverage. We have excluded patients residing outside the city boundaries to increase the likelihood that temporal variation in exposure corresponded to the measured temporal variation of air pollution, but some misclassification of exposure might have occurred. Third, because of the large number of model runs (3 pollutant × 3 lags), it is possible that some significant results might happen by chance only. Finally, the case-only analysis was based on a subset of total AF records coming from a single hospital and for a restricted time period, and the statistical power of this analysis was possibly lower for medical conditions with small occurrence in the sample.

## 5. Conclusions

Arrhythmias, of which AF is the most prevalent form, have been linked with major cardiovascular events such as ischemic stroke and heart failure. The deregulation of the autonomic nervous system and/or the inflammatory response are known causes of AF onset and are linked to pulmonary oxidative stress and inflammation following exposure to high levels of air pollutants. Our results add evidence to the fact that a high concentration of air pollution is associated with an increased number of people seeking emergency aid because of AF within 24 h. The effect of increased PM_10_ and NO_2_ on AF admission was exacerbated in individuals with a previous history of CVD. Since people with AF have a 5-fold increased risk of stroke [56], even a modest risk associated with exposure to high levels of air pollution would largely increase the attributable risk in the general population.

## Figures and Tables

**Table 1:** Summary statistics of daily exposure and number of emergency visits for atrial fibrillation (AF) in Rome in the 2001–2014 period.

Variables	N (Days)	Mean	SD	Min	Max
PM_10_	5111	34.5	15.1	4.0	181.7
PM_2.5_	3272	18.7	10.0	0.0	72.6
NO_2_	5110	58.3	16.9	11.9	117.9
Temperature	5041	15.9	7.0	−1.0	31.0
Relative humidity	4360	74.4	12.1	31.0	98.6
Emergency visits for AF	5113	15.6	4.9	1.0	36.0

**Table 2:** Association between AF emergency visits and air pollution levels in fully adjusted models. Reported statistics are the percent increase in the risk of AF admission per 10 µg/m^3^ increase of pollutant (95% CI).

Pollutant	Selection	Immediate (Lag 0–1)	Delayed (Lag 2–5)	Extended (Lag 0–5)
PM_10_	All	1.44 (0.65, 2.26) *	−0.04 (−0.89, 0.81)	0.70 (−0.30, 1.72)
	<75 years	0.66 (−0.39, 1.72)	−0.12 (−1.24, 1.02)	0.26 (−1.07, 1.60)
	≥75 years	2.70 (1.42, 4.07) *	−0.04 (−1.43, 1.38)	1.39 (−0.27, 3.09)
	Men	1.51 (0.15, 2.91) *	1.17 (−0.29, 2.65)	1.87 (0.15, 3.64) *
	Women	1.15 (−0.13, 2.47)	−1.28 (−2.63, 0.11)	−0.57 (−2.18, 1.06)
PM_2.5_	All	2.95 (1.35, 4.67) *	0.13 (−1.49, 1.78)	1.70 (−0.29, 3.76)
	<75 years	1.44 (−0.63, 3.56)	−0.45 (−2.52, 1.67)	0.43 (−2.10, 3.03)
	≥75 years	5.01 (2.59, 7.74) *	0.93 (−1.53, 3.47)	3.43 (0.42, 6.66) *
	Men	2.31 (−0.02, 4.75)	0.48 (−1.87, 2.90)	1.59 (−1.27, 4.56)
	Women	3.51 (1.29, 5.90) *	−0.18 (−2.38, 2.08)	1.77 (−0.96, 4.61)
NO_2_	All	1.19 (0.27, 2.13) *	0.44 (−0.57, 1.47)	1.10 (−0.11, 2.34)
	<75 years	1.09 (−0.12, 2.33)	0.09 (−1.25, 1.44)	0.70 (−0.89, 2.33)
	≥75 years	1.50 (0.03, 3.02) *	0.73 (−0.90, 2.39)	1.57 (−0.37, 3.57)
	Men	1.17 (−0.39, 2.77)	1.83 (0.10, 3.62) *	2.40 (0.35, 4.55) *
	Women	0.89 (−0.58, 2.40)	−1.12 (−2.73, 0.52)	−0.54 (−2.47, 1.43)

* *p*-value < 0.05.

**Table 3:** Effect modification of pre-existing medical conditions on the effect of PM_10_, PM_2.5_, and NO_2_ on AF emergency hospital visits. Results are expressed as interaction rate ratios (IRRs) per 10 μg/m^3^ increase of pollutant.

Pollutant	Lag	Diabetes	Cardiovascular Disease	Chronic Respiratory Disease	Oncological, Liver, Renal, or Neurodegenerative Disease
PM_10_	0–1	0.98 (0.91, 1.06)	1.06 (1.00, 1.12) *	0.97 (0.89, 1.05)	0.99 (0.94, 1.05)
	2–5	1.04 (0.96, 1.13)	1.07 (1.00, 1.14) *	1.01 (0.92, 1.11)	0.97 (0.91, 1.04)
	0–5	1.02 (0.93, 1.12)	1.09 (1.01, 1.17) *	0.99 (0.89, 1.10)	0.97 (0.91, 1.05)
PM_2.5_	0–1	0.93 (0.80, 1.07)	1.08 (0.98, 1.19)	0.99 (0.85, 1.15)	1.03 (0.93, 1.14)
	2–5	1.02 (0.88, 1.18)	1.05 (0.95, 1.17)	0.97 (0.82, 1.14)	0.99 (0.89, 1.11)
	0–5	1.00 (0.84, 1.19)	1.10 (0.97, 1.25)	0.96 (0.79, 1.16)	1.03 (0.90, 1.17)
NO_2_	0–1	0.99 (0.92, 1.06)	1.07 (1.01, 1.13) *	1.06 (0.98, 1.15)	0.98 (0.93, 1.04)
	2–5	1.00 (0.93, 1.08)	1.06 (1.00, 1.12) *	1.03 (0.94, 1.13)	0.97 (0.92, 1.03)
	0–5	0.99 (0.91, 1.08)	1.08 (1.01, 1.15) *	1.05 (0.96, 1.16)	0.97 (0.91, 1.04)

* *p*-value < 0.05.

## Supplementary Materials

The following are available online at www.mdpi.com/1660-4601/14/6/661/s1, Figure S1: Relative risks of emergency room admission for atrial fibrillation associated with PM_10_ of 44.5 µg/m^3^ compared to a centered concentration of PM_10_ = 34.5 µg/m^3^ (mean of the whole study period) on lag 0 to 5 in Rome during 2001–2014, Figure S2: Relative risks of emergency room admission for atrial fibrillation associated with PM_2.5_ of 28.7 µg/m^3^ compared to a centered concentration of PM_2.5_ = 18.7 µg/m^3^ (mean of the whole study period) on lag 0 to 5 in Rome during 2001–2014, Figure S3: Relative risks of emergency room admission for atrial fibrillation associated with NO_2_ of 68.3 µg/m^3^ compared to a centered concentration of NO_2_ = 58.3 µg/m^3^ (mean of the whole study period) on lag 0 to 5 in Rome during 2001–2014, Table S1: Bipollutant models of the association between AF emergency hospital admissions and air pollution levels. Reported statistics are the % increase in risk per 10 unit increase of pollutant (95% CI), Table S2: Pre-existing medical conditions in the consecutive case series coming from a single hospital from which those data were available (*n* = 4482; only years 2006–2010). Median age was 72 years (min = 45; max = 100); women represented 51.8% of total patients.
