# Supplementary material for: Association between Air Pollution and Emergency Room Visits for Atrial Fibrillation
Source: Int J Environ Res Public Health. 2017 Jun 20;14(6):661. doi: 10.3390/ijerph14060661 (PMC5486347; doi:10.3390/ijerph14060661)
Supplement: Supplementary file 1 [file ijerph-14-00661-s001.pdf]

Association between Air Pollution and Emergency Room Visits for Atrial Fibrillation

Table S1. Bipollutant models of the association between AF emergency hospital admissions and air pollution levels. Reported statistics are the % increase in risk per 10 unit increase of pollutant (95% CI).

	Immediate (Lag 0–1)	Retarded (Lag 2–5)	Extended (Lag 0–5)
PM_{10} with NO_2	1.23 (0.31, 2.16)*	−0.38 (−1.41, 0.67)	0.27 (−0.94, 1.5)
$PM_{2.5}$ with NO_2	2.04 (0.26, 3.90)*	−0.30 (−2.46, 1.14)	0.69 (−1.89, 3.34)
NO_2 with PM_{10}	0.50 (−0.54, 1.56)	0.71 (−0.54, 1.97)	0.92 (−0.55, 2.41)
NO_2 with PM_{25}	1.25 (−0.18, 2.71)	0.52 (−1.22, 2.29)	1.24 (−0.80, 3.33)

* p-value < 0.05.

Table S2. Pre-existing medical conditions in the consecutive case series coming from a single hospital from which those data were available (n = 4482; only years 2006–2010). Median age was 72 years (min = 45; max = 100); women represented 51.8% of total patients.

Medical condition	Frequency (n = 4482)
Diabetes	11.9%
Cardiovascular disease	75.4%
Chronic respiratory disease	8.8%
Oncological, liver, renal or neurodegenerative disease	22.5%

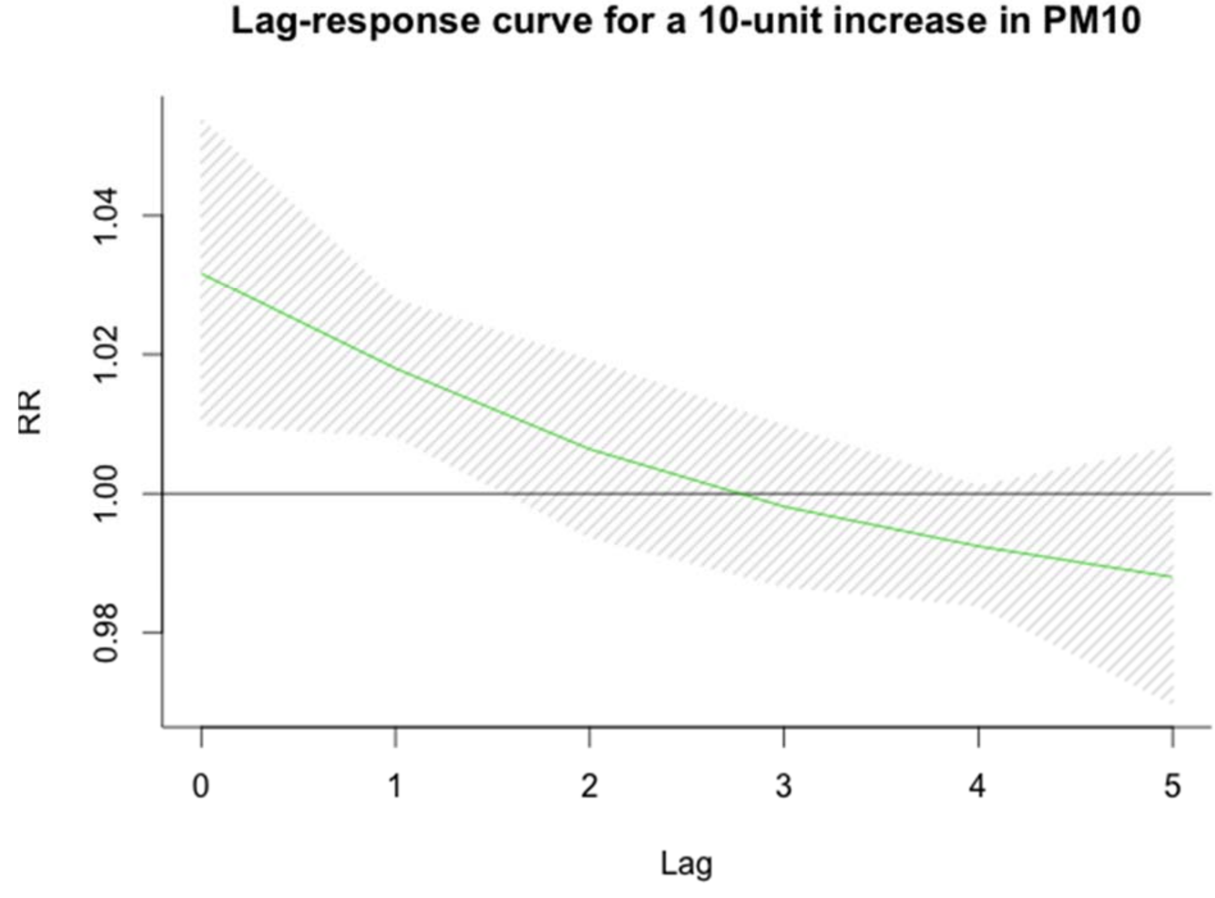

Figure S1. Relative risks of emergency room admission for atrial fibrillation associated with PM_{10} of 44.5 μg/m^3 compared to a centered concentration of PM_{10} = 34.5 μg/m^3 (mean of the whole study period) on lag 0 to 5 in Rome during 2001–2014.

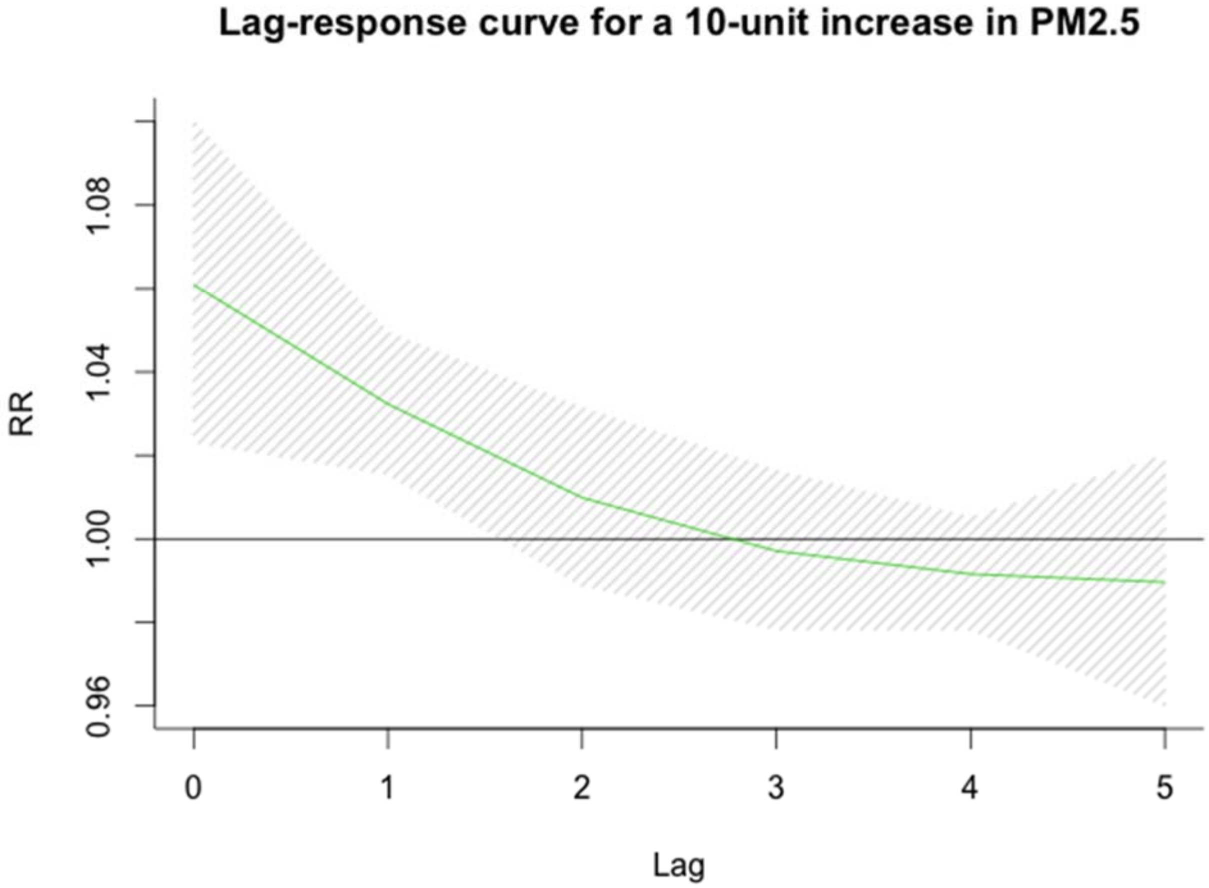

Figure S2. Relative risks of emergency room admission for atrial fibrillation associated with $PM_{2.5}$ of 28.7 μg/m^3 compared to a centered concentration of $PM_{2.5}$ = 18.7 μg/m^3 (mean of the whole study period) on lag 0 to 5 in Rome during 2001–2014.

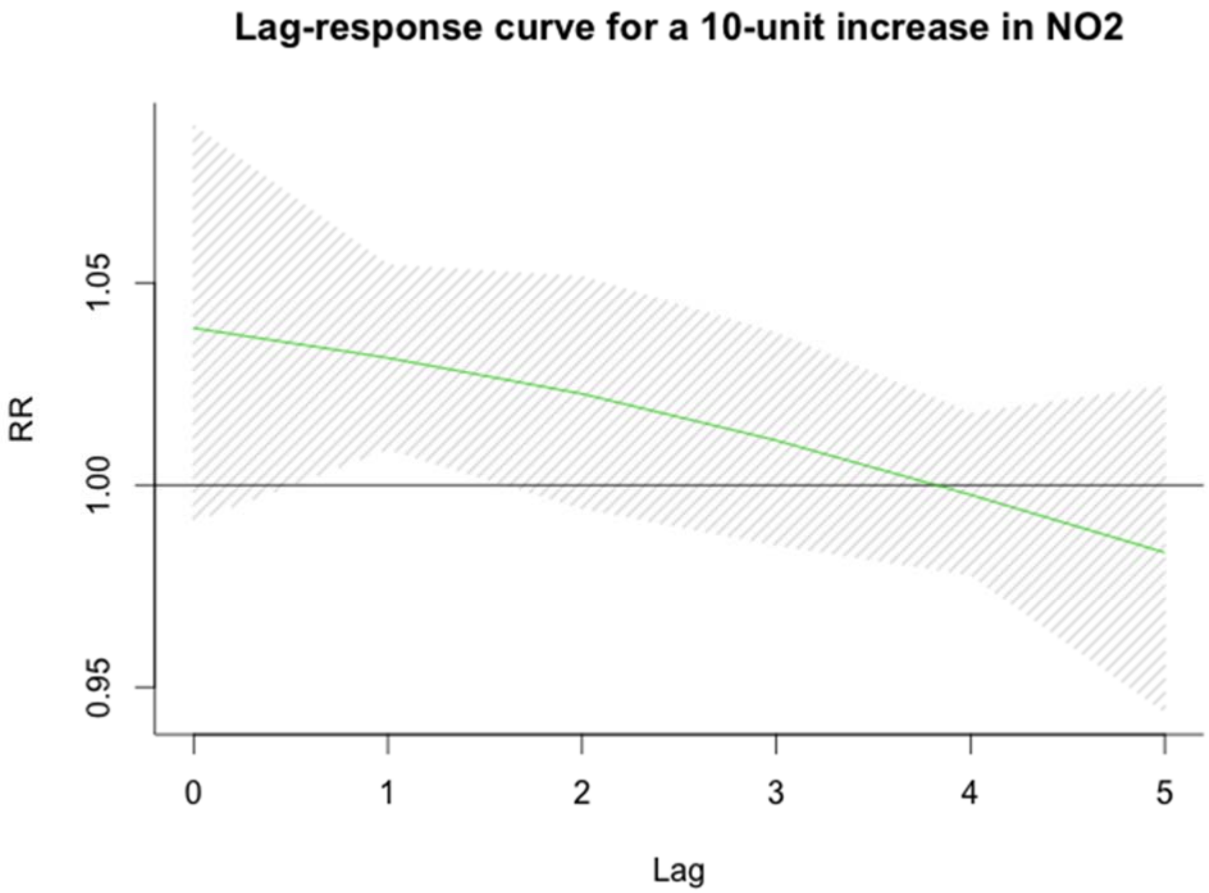

Figure S3. Relative risks of emergency room admission for atrial fibrillation associated with NO_2 of 68.3 μg/m^3 compared to a centered concentration of NO_2 = 58.3 μg/m^3 (mean of the whole study period) on lag 0 to 5 in Rome during 2001–2014.
